# User Satisfaction With a Daily Supportive Text Message Program (Text4PTSI) for Public Safety Personnel: Longitudinal Cross-Sectional Study

**DOI:** 10.2196/46431

**Published:** 2023-06-23

**Authors:** Gloria Obuobi-Donkor, Ejemai Eboreime, Reham Shalaby, Belinda Agyapong, Natalie Phung, Scarlett Eyben, Kristopher Wells, Raquel da Luz Dias, Carla Hilario, Chelsea Jones, Suzette Brémault-Phillips, Yanbo Zhang, Andrew J Greenshaw, Vincent Israel Opoku Agyapong

**Affiliations:** 1 Department of Psychiatry, Faculty of Medicine Dalhousie University Halifax, NS Canada; 2 Department of Psychiatry, Faculty of Medicine and Dentistry University of Alberta Edmonton, AB Canada; 3 Operational Stress Injury Clinic Alberta Health Services Edmonton, AB Canada; 4 Department of Child and Youth Care Faculty of Health and Community Studies MacEwan University Edmonton, AB Canada; 5 Faculty of Nursing University of Alberta Edmonton, AB Canada

**Keywords:** public safety personnel, Text4PTSI, text messaging, satisfaction, occupational health, work safety, public safety, digital health intervention, mental health service, user satisfaction

## Abstract

**Background:**

Public safety personnel (PSP) are exposed to traumatic events due to their work environments, which increases the risk of mental health challenges. Providing effective and evidence-based interventions, such as SMS text messaging programs, can improve PSP's overall mental well-being with high user satisfaction rates.

**Objective:**

This study aims to evaluate users’ satisfaction, receptiveness, and perceptions of a cognitive behavioral therapy (CBT)–based supportive SMS text messaging intervention (Text4PTSI).

**Methods:**

Participants self-subscribed to Text4PTSI and received unidirectional cognitive behavioral–based supportive text messages for 6 months. Participants completed a web-based survey delivered via SMS text message at enrollment, and 6 weeks, 3 months, and 6 months post enrollment. Respondents’ perception and receptivity of the program were assessed using a questionnaire measured on a 5-point Likert scale. Data were collected as categorical variables, and overall satisfaction with the Text4PTSI program was measured on a scale from 0 to 100.

**Results:**

There were 131 subscribers to the Text4PTSI program; however, only 81 subscribers responded to the survey, producing 100 survey responses across the 3 follow-up time points. The overall mean score of satisfaction was 85.12 (SD 13.35). More than half of the survey responses agreed or strongly agreed that Text4PTSI helped participants cope with anxiety (79/100 responses, 79%), depressive symptoms (72/100 responses, 72%), and loneliness (54/100 responses, 54%). Similarly, most of the survey responses agreed or strongly agreed that the Text4PTSI program made respondents feel connected to a support system, improved their overall mental well-being (84/100 responses, 84%), felt more hopeful about managing concerns about their mental health or substance use (82 out of responses, 82%), and helped enhance their overall quality of life (77/100 responses, 77%). The available survey responses suggest that the majority always read the supportive text messages (84/100 responses, 84%), took time to reflect on each message (75/100 responses, 75%), and returned to read the text messages more than once (76/100 responses, 76%).

**Conclusions:**

PSP who responded to the follow-up surveys reported high user satisfaction and appreciation for receiving the Text4PTSI intervention during the 6-month program. The reported satisfaction with the service provided could pave the way to ensuring a better uptake of the service with potential effectiveness to end users.

## Introduction

Mental well-being is an important issue for public safety personnel (PSP) in Canada, as these individuals often experience high levels of stress and psychological trauma due to their occupations. The definition of PSP advancing includes but is not limited to the paramedic, police, correctional workers, firefighters, and all first responder groups [1]. The stressors at work can lead to various mental health challenges, including depression, anxiety, and posttraumatic stress disorder [2-5].

The challenging and complex nature of PSP’s work is one of the main sources of stress. These individuals are often exposed to traumatic events, such as accidents, natural disasters, and violent crimes [2-5]. These often unpredictable events can significantly negatively affect their mental health, as they may struggle to cope with the emotional impact of these often psychologically traumatic and morally ambiguous events. Additionally, PSP often work long and irregular hours, making it challenging to maintain a healthy lifestyle and work-life balance. Another source of stress for PSP may include the culture of their organizations [6]. Many public safety agencies have a culture of stoicism and toughness, which can discourage employees from seeking help for mental health problems. The workplace environment can make it difficult for PSP to seek out the necessary support they need to cope with the stress and psychological trauma of their work [2-5].

A study among Canadian PSP revealed that about 44.5% self-reported at least 1 mental disorder [7,8]. To address these issues, several initiatives have been aimed at promoting the mental well-being of PSP in Canada. Some of these programs provide training and resources to help PSP cope with the stress and psychological trauma of their work [9]. Additionally, many public safety agencies have employee assistance programs that provide confidential counseling and support to employees struggling with mental health concerns [10].

However, despite these supportive efforts, there is still work to be done to improve the mental well-being of PSP in Canada. The Canadian Institute for Public Safety Research and Treatment reports that 44.5% of PSP in Canada screened positive for 1 or more mental health symptoms [11]. Some of the gaps relate to these personnel’s mental health care–seeking behavior. Another study identified how PSP are hesitant to access mental health services [12]. Despite attempts to reduce the barriers experienced by PSP when they are accessing mental health services, stigma and geographical barriers inhibit demand for and success to care [8].

While several interventions for PSP, ranging from pharmacology to psychotherapies, have been described in the research literature [13], it is essential to deliver accessible, effective, and economic remote access technologies such as supportive text messages, which are evidence-based and practical techniques for reducing and managing mental health conditions [14-21]. The usage of mobile technologies in mental health is relatively new and novel, although other areas of health practice have used text messaging systems [20,22,23]. In community-based studies, individuals with severe mental health conditions possess mobile phones and are willing to use the telephone for recovery support [24,25]. Additionally, interventions delivered via telephone show reduced attrition compared to face-to-face programs, which reduces geographical barriers [26].

When supportive intervention is provided, most studies have demonstrated a decrease in mental health symptoms. For example, in a longitudinal study to assess the effectiveness of an SMS text message intervention, PSP who subscribed to the texting program reported that they were able to manage depression, anxiety, and other psychological symptoms [6]. Similarly, 10 randomized controlled trials providing therapeutic intervention through texting demonstrated a substantial improvement in psychological symptoms compared to conventional treatment [27]. A qualitative analysis of a stakeholder perspectives intervention for PSP revealed that they believe that an internet cognitive behavioral therapy (iCBT) intervention is an appropriate treatment option to address mental health issues among Canadian PSP [28]. In addition, the findings from supportive text messaging programs like Text4Hope and Text4Mood suggested that mental health symptoms among respondents were minimized after receiving the supportive texts compared to respondents’ baseline scores on the self-reported validated scales [14,20]. Beyond the effectiveness of these interventions, services must also be acceptable to those who use them [29]. User satisfaction is known to affect client retention and clinical outcomes of interventions [30]. In this study, we evaluate the satisfaction of PSP with Text4PTSI, an evidence-based intervention to improve the mental well-being and provide support to those experiencing PTSI.

We hypothesize that participants who subscribe to the Text4PTSI program will have high user satisfaction, positive program perceptions, and receptivity, which will remain stable over time. Hence, we propose the following research questions: (1) how satisfied are subscribers with the Text4PTSI program for 6 weeks, 3 months, and 6 months? (2) what are the participants’ perceptions of the Text4PTSI program at 6 weeks, 3 months, and 6 months? and (3) how are the satisfaction rates and subscriber perception of Text4PTSI similar or dissimilar across the 3 time points?

## Methods

### Study Design

This research was designed as a longitudinal cross-sectional study, which assessed participants’ satisfaction and experiences with the Text4PTSI program at 6 weeks, 3 months, and 6 months post intervention. The sample reported in this study is less than anticipated since PSPs who subscribed to the Text4PTSI program were less than 10% of the projected 5000 subscribers indicated in the published protocol.

### Text4PTSI Program and Data Collection

Text4PTSI was launched in July 2021 to support PSP’s mental health in Alberta, Canada [31,32]. The targeted PSP group included emergency department health workers, paramedics, and police and law enforcement agents. This innovative program provides supportive text messages to PSP to prevent and manage posttraumatic stress disorder and other common mental health symptoms. The text messages are based on CBT principles [33]. The messages had a 160-character limit written by cognitive behavioral therapists in partnership with patients and other mental health professionals who addressed the aspects of potential psychological stress, anxiety, and depression. Examples of the text messages sent are:

Trauma can feel like a gloomy cloud over all areas of your life. The first step in treatment is to understand what trauma is, the symptoms, and how and why it is treated.

If you find yourself worrying excessively, make some boundaries with these thoughts. Jot down your worries throughout the day and let them go. Then set a time aside for 20 minutes to review what you wrote down.

Similar programs have demonstrated effectiveness in mitigating mental health symptoms like mood disorders in the general population with high user satisfaction rates. For example, a study on the impact of supportive messages among subscribers of Text4Mood reported that about 82% of the respondents felt hopeful about managing life issues and experienced an overall mental well-being [20].

Data collection procedures used in this study are described in the published study protocol [33]. In summary, PSPs enroll in the program by texting “*PTSI*” to 1-844-990-4343. From the day of enrollment, participants receive 1 text daily, which is unidirectional computer-programmed supportive text messages. These messages are based on CBT principles. Users are at an advantage similar to CBT sessions, where a therapist helps participants to evaluate and challenge negative thoughts, feelings, and emotions [32]. Participants are also invited to complete a web-based program evaluation at 6 weeks, 3 months, and 6 months after enrolling in the intervention. The survey generally took 5 to 10 minutes to complete using their cell phones.

Participation in the Text4PTSI program was voluntary, with no incentives. Receiving supportive messages was independent of survey completion. Participants could opt out of the program at any time by texting back the word “*STOP.*”

**Figure 1:**
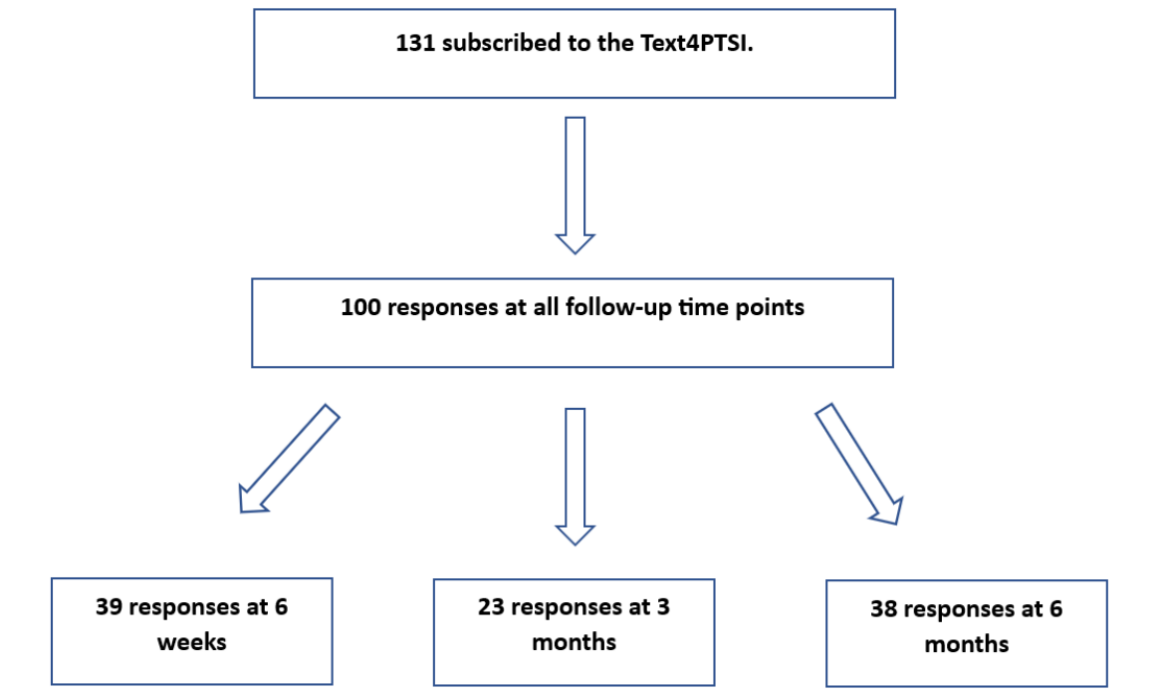
Study flowchart.

Satisfaction data were collected between September 7, 2021, and October 27, 2022. Figure 1 illustrates the participants' pathways when completing the surveys at each designated time point.

### Ethics Approval

The Text4PTSI study received ethical approval from the University of Alberta Health Research Ethics Board (Pro00108966) [33]. Informed consent was implied by all study participants after they had read the web-based information leaflet, completed the web-based survey, and returned their responses.

### Outcome Measures

The primary outcome measure was the participants’ overall satisfaction with the Text4PTSI program, which delivered to subscribers daily supportive text messages designed to support mental health. Responses were calculated by using subscribers’ responses to 1 question “Using any number from 0 (not at all satisfied) to 100 (very satisfied), how would you rate your overall satisfaction with Text4PTSI?”

Secondary outcomes included the perceived impacts of the program on participants’ mental health and feedback of the supportive text messages at 6 weeks, 3 months, and 6 months from enrollment. Text4PTSI participants provided feedback on the text messaging intervention by responding to questions assessing the following two focal areas:

1. Perception on how participants coped with stress, anxiety, and depression; connection to a support system; quality of life; and overall mental wellness after receiving the daily supportive text messages (6 weeks, 3 months, and 6 months): The questionnaire was measured on a 5-point Likert scale; agree, strongly agree, neutral, disagree, and strongly disagree. For the purpose of analyses, responses were collapsed into 3 points: agree or strongly agree, neutral, and disagree or strongly disagree.
2. Receptivity of the Text4PTSI program: These questionnaires were assessed on a 5-point Likert scale: always, often, sometimes, rarely, and never.

Although reliability and validity measures for the satisfaction scale have not been assessed, the survey questions have been used in previous studies to evaluate user satisfaction with similar supportive text messaging programs [20,34].

### Data Analyses

Data were analyzed using SPSS (version 25; IBM Corp) statistical software for Windows [35]. Participants’ satisfaction data were presented as a continuous variable. We measured participants’ overall satisfaction by rating the program from 0 to 100 (0=very dissatisfied, 50=neutral, and 100=very satisfied). The data were reported as means and SDs.

We summarized the satisfaction and participants’ perception of the program at different time points (6 weeks, 3 months, and 6 months). Satisfaction data were presented as categorical variables and reported as frequency and percentages across all the study participants at all 3 time points. The average percentage of all 3 time points was then tabulated and reported.

A chi-square analysis was run to examine any differences in reporting satisfaction across the 3 time points. There was no imputation for missing data, and the results were based on completed survey responses.

## Results

### Overview

As illustrated in Figure 1, participating individuals who subscribed to the Text4PTSI program were 131 which produced 100 usable individual survey responses across the 3 follow-up time points (39 at 6 weeks, 23 at 3 months, and 38 at 6 months), of which 81 respondents completed the survey. Since the expectation was that 131×3 respondents would respond to the survey at the 3 time points, the response rate yielded in this study was 25.4% (100/393). Reported demographic data are provided in Table 1. Police and law enforcement agents were the majority of respondents, followed by female (47/81, 58%), White (63/81, 78%), obtained postsecondary education (68/81, 84%), employed (71/81, 88%), in a relationship (58/81, 73%), and own home (57/81, 70%). The majority of the subscribers were between 31 and 45 years of age.

**Table 1:** Demographic data of study subscribers.

Variable^a^		Participants, n (%)
**Public safety personnel**		
	Emergency department health workers	8 (12)
	Paramedics	19 (30)
	Police and law enforcement agents	30 (47)
	Other	7 (11)
**Age groups (years)**		
	≤30	14 (18)
	31-45	31 (39)
	46-60	25 (31)
	>60	10 (12)
**Gender**		
	Female	47 (58)
	Male	31 (38)
	Nonbinary	3 (4)
**Ethnicity**		
	White	63 (78)
	Non-White	18 (22)
**Education level**		
	Postsecondary education	68 (84)
	Other	13 (16)
**Employment status**		
	Employed	71 (88)
	Unemployed	10 (12)
**Relationship status**		
	In a relationship	58 (73)
	Not in a relationship	22 (27)
**Housing status**		
	Own a home	57 (70)
	Renting	17 (21)
	Living with family	7 (9)

^a^Not all participants provided a response.

### Participants’ Satisfaction, Perceptions, and Receptivity

Participants rated the program by indicating their overall satisfaction on a scale of 0-100, with zero indicating very dissatisfied, 50 indicating neutral (neither satisfied nor dissatisfied), and 100 representing very satisfied. The total number of participants providing their feedback was 100, with an overall satisfaction mean score of 85.12 (SD 13.35). The descriptive analysis also recorded a mean 95% CI of 82.5-87.8. This result indicates that, overall, respondents were satisfied with the Text4PTSI program.

Table 2 represents the level of agreement regarding the Text4PTSI program and its benefits to users. Table 2 indicates 79 out of 100 (79%) survey responses agreed or strongly agreed that the supportive text messages helped cope with anxiety, 72 responses (72%) reported the messages helped them cope with depression and 54 (54%) reported the messages helped them cope with loneliness. In addition, 84 out of 100 responses (84%) agreed or strongly agreed the Text4PTSI program made participants feel connected to a support system and improved their overall mental well-being. Overall, 82 out of 100 responses (82%) indicated they felt hopeful about managing concerns with their mental health or substance use; and 77 out of 100 responses (77%) agreed or strongly agreed that supportive text messages enhanced their quality of life.

Table 3 shows participants’ perceptions of the supportive text message program 6 weeks, 3 months, and 6 months post intervention. Overall, some of the responses suggested SMS text messages were always positive, affirmative, and succinct. The supportive text messages were always inclusive and relevant to participants’ identity background (eg, age, gender, ethnicity, sexual orientation, and gender identity). Table 3 suggests that more than half of the respondents always read the SMS text messages and took time to reflect on the message while indicating and receiving the supportive text messages once daily. In comparison, 76 out of 100 responses (76%) suggested that at least sometimes they return to read the supportive text messages more than once. The supportive text messages were delivered once daily; 93 out of 100 responses (93%) indicated the majority of participants were satisfied with the frequency.

Overall, the chi-square analysis revealed no significant difference in reporting the different satisfaction items among the 3 time points.

**Table 2:** Perceived impact of daily messages postintervention at all 3 time points.

Perceived impact of Text4PTSI			Six weeks, n (%)		Three months, n (%)		Six months, n (%)		Total, n (%)
**Helped participants to cope with anxiety**									
	Agree or strongly agree	32 (82)		18 (78)		29 (76)		79 (79)	
	Neutral	6 (15)		5 (22)		9 (24)		20 (20)	
	Disagree or strongly disagree	1 (3)		0 (0)		0 (0)		1 (1)	
**Helped participants to cope with depression**									
	Agree or strongly agree	25 (64)		17 (74)		30 (79)		72 (72)	
	Neutral	12 (31)		5 (22)		7 (18)		24 (24)	
	Disagree or strongly disagree	2 (5)		1 (4)		1 (3)		4 (4)	
**Helped participants to cope with loneliness**									
	Agree or strongly agree	20 (51)		14 (61)		20 (53)		54 (54)	
	Neutral	16 (41.)		7 (30)		11 (29)		34 (34)	
	Disagree or strongly disagree	3 (8)		2 (9)		7 (18)		12 (12)	
**Participants felt connected to a support system**									
	Agree or Strongly agree	34 (87)		18 (78)		32 (84)		84 (84)	
	Neutral	4 (10)		5 (22)		4 (11)		13 (13)	
	Disagree or strongly disagree	1 (3)		0 (0)		2 (5)		3 (3)	
**Helped participants feel hopeful to manage mental health or substance** **use concerns**									
	Agree or strongly agree	32 (82)		19 (83)		31 (82)		82 (82)	
	Neutral	7 (18)		4 (17)		7 (18)		18 (18)	
**Helped participants improve their overall mental well-being**									
	Agree or Strongly agree	30 (77)		20 (87)		34 (89)		84 (84)	
	Neutral	9 (23)		2 (9)		3 (8)		14 (14)	
	Disagree or strongly disagree	0 (0)		1 (4)		1 (3)		2 (2)	
**Helped participants enhance their quality of life**									
	Agree or strongly agree	29 (74)		17 (74)		31 (82)		77 (77)	
	Neutral	9 (23)		5 (22)		7 (18)		21 (21)	
	Disagree or strongly disagree	1 (3)		1 (4)		0 (0)		2 (2)	

**Table 3:** Participants’ feedback 6 weeks, 3 months, and 6 months after the intervention.

Feedback			Six weeks, n (%)		Three months, n (%)		Six months, n (%)		Total, n (%)
**The Text4Hope text messages were positive**									
	Always	26 (67)		15 (65)		25 (66)		66 (66)	
	Often	11 (28)		8 (35)		13 (34)		32 (32)	
	Sometimes	1 (3)		0 (0)		0 (0)		1 (1)	
	Rarely	1 (3)		0 (0)		0 (0)		1 (1)	
**The Text4Hope-Addiction text messages were affirmative**									
	Always	25 (64)		15 (65)		25 (66)		65 (65)	
	Often	12 (31)		8 (35)		12 (31)		32 (32)	
	Sometimes	1 (3)		0 (0)		1 (3)		2 (2)	
	Rarely	1 (3)		0 (0)		0 (0)		1 (1)	
**The Text4Hope-Addiction text messages were succinct**									
	Always	23 (59)		13 (57)		22 (58)		58 (58)	
	Often	12 (31)		10 (43)		14 (37)		36 (36)	
	Sometimes	3 (8)		0 (0)		2 (5)		5 (5)	
	Rarely	1 (3)		0 (0)		0 (0)		1 (1)	
**Inclusive of and relevant to my identity background**									
	Always	24 (61)		15 (65)		21 (55)		60 (60)	
	Often	11 (28)		8 (35)		14 (37)		33 (33)	
	Sometimes	3 (8)		0 (0)		3 (8)		6 (6)	
	Never	1 (3)		0 (0)		0 (0)		1 (1)	
**Frequency reading the Text4PTSI text messages**									
	Always	31 (79)		20 (87)		33 (87)		84 (84)	
	Often	7 (18)		3 (13)		4 (10)		14 (14)	
	Sometimes	1 (3)		0 (0)		1 (3)		2 (2)	
**Action taken after reading text messages**									
	Read the text and took no action	6 (15)		2 (9)		3 (8)		11 (11)	
	Read the text and took time to reflect on the message	26 (67)		17 (74)		32 (84)		75 (75)	
	Read the text and took a positive or beneficial action	7 (18)		4 (17)		3 (8)		14 (14)	
**Return to reading Text4PTSI text messages more than once**									
	Always	3 (8)		1 (4)		4 (10)		8 (8)	
	Often	7 (18)		5 (22)		11 (29)		23 (23)	
	Sometimes	17 (44)		10 (44)		18 (47)		45 (45)	
	Rarely	10 (25)		4 (17)		4 (10)		18 (18)	
	Never	2 (5)		3 (13)		1 (3)		6 (6)	
**Satisfaction with the frequency of the Text4PTSI text messages**									
	Satisfied	34 (87)		22 (96)		37 (97)		93 (93)	
	Neutral	5 (13)		1 (4)		1 (3)		7 (7)	
**Frequency participants prefer to receive supportive text messages**									
	Twice daily	2 (5)		2 (9)		4 (11)		8 (8)	
	Once daily	30 (77)		16 (70)		29 (76)		75 (75)	
	Once every other day	5 (13)		4 (17)		2 (5)		11 (11)	
	Once weekly	2 (5)		1 (4)		3 (8)		6 (6)	

## Discussion

### Principal Findings

This study presents novel findings on how PSP perceived daily supportive text messages and their receptiveness to the Text4PTSI program. The results from the study indicated high respondents’ satisfaction with the program, with a mean score of 85.12 (SD 13.35) that is similar to other studies [34,36]. Overall, the majority of survey responses agreed or strongly agreed with the intervention’s positive effect in helping participants cope with anxiety, depressive symptoms, and loneliness and feel connected to a support system postintervention. The Text4PTSI program helped most respondents feel hopeful about managing their mental health or substance use concerns, improving their overall mental well-being and quality of life.

The survey responses suggested participants were satisfied with the frequency of the messages, and 3 in 4 (75%) responses suggested participants would prefer to receive supportive text messages once daily. Similarly, the majority of responses reported that participants perceived supportive text messages as always positive, affirmative, and succinct. In addition, the supportive text messages were reported as always inclusive and relevant to 60 (60%) of the respondents’ identity background (eg, age, gender, ethnicity, sexual orientation, and gender identity), while respondents always or often read the messages and took time to reflect on the messages.

Reported satisfaction with the Text4PTSI program aligns with satisfaction with other supportive text messaging programs geared toward improving the mental health of subscribers [20,34,37]. For example, an SMS text messaging intervention program delivering a daily supportive text message during the COVID-19 pandemic reported a mean overall participant satisfaction of 8.55 [34], which is consistent with the findings from this study. Similarly, after 12 weeks of a text messaging intervention for smoking cessation, nearly 91% (40/44) of the participants expressed high satisfaction with the program [36]. These findings suggest that respondents are satisfied when supportive messages are incorporated into their care.

High satisfaction with the Text4PTSI intervention implies respondents benefit from the program and highly recommend Text4PTSI to help broadly manage the mental health burden of PSP. A study among first responders, which focused on the usage of telehealth care, reported that out of 38 individuals who were recommended to seek mental health services, about 29 (76%) agreed to adhere to treatment via web-based or telehealth means [38].

PSP often benefit from evidence-based iCBT to assist with mental health symptoms, process emotions, and address potentially traumatic or morally distressing events. Qualitative research reveals that PSP positively perceives iCBT intervention to manage their mental health concerns [28].

Our study results aligned with similar studies focused on other PSP or who use a similar texting service. Another program providing supportive care based on iCBT revealed similar results among PSP [39]. The research team reported that 87% (54/62) of PSP who participated in the iCBT program experienced high confidence in managing their mental health symptoms. Almost all 98% (61/62) agreed that the program was effective [39]. Consistent with the later study, the findings from this study demonstrated how most participants agreed that Text4PTSI helped them to cope with various mental health symptoms albeit with lower proportions. This study’s results are also slightly higher than those of a similar program [34]. The Text4Hope program, which delivered supportive interventions to individuals through texting, reported that, after 6 weeks of providing the intervention, 76% of their study sample reported that they could cope with their anxiety symptoms, 56% could cope with any depression symptoms, while 49% were able to cope with loneliness [34]. Furthermore, the effectiveness of Text4PTSI improved participants’ overall well-being, and participants reported improved quality of life with a follow-up prevalence. Supportive texting programs like Text4PTSI support the growing evidence indicating that iCBT and a supportive text message intervention can potentially improve participants’ addiction, mental health, and quality of life for most PSP [39-41]. Hence, when these supportive messages are delivered to individuals, the messages have the prospect of improving overall well-being.

Text messaging technology can help improve access to health care in an era of inadequate health resources. Six months after the Text4PTSI program, 87.2% of study responses agreed or strongly agreed that they felt connected to a support system. This result aligns with the research literature indicating how supportive text messages can expand one’s perceived support network and increase personal support systems [41]. The findings also correspond with the belief that text message technology has the prospect of delivering remote health care to people [37,42]. This result may reflect PSPs need to connect with a health care system to bridge the gap in using mental health support and services. Notwithstanding, individuals who do not sign up for such supportive text interventions may not benefit.

Recent studies indicate how mental health interventions delivered via mobile phones are positive, affirmative, and relevant [20,34,43,44]. In research where supportive text messages were delivered to manage drug addiction, users felt the messages were positive and positively impacted their way of life [45]. Consistent with our study, 99% of respondents agreed that the content of the supportive text messaging was inclusive of and relevant to their identity background and the supportive text messages addressed potential differences in age, gender, ethnicity, and gender identity. Hence, respondents were satisfied with the SMS text messages they received. These results reflect why study participants agree text messages are positive, affirmative, and relevant sources of mental health support and when these interventions are continuously delivered will positively impact the mental health of individuals.

This study included questions about how participants return to read, reflect, and take action after reading the supportive text messages they received. Reflective and impulsive processes are correlated and intentionally help individuals identify their intentions and activities and develop coping skills [46]. Across the follow-up postintervention, our study recorded a consistent increase in the positive action taken after reading supportive text messages. At 6 weeks, 3 months, and 6 months, 66.7%, 73.9%, and 84.2% of the respondents, respectively, read the text and took time to reflect on the message, while 14% read and took positive action. This is consistent with a similar study from Alberta, Canada, which provided supportive text messages primarily to patients with depressive or anxiety symptoms and others [20]. Another study providing supportive text messages during the pandemic reported that 76% of respondents read and reflected on the text [34]. In a randomized controlled trial to assess the feasibility of health intervention delivered through email or text messages, results revealed that 83% of participants in the text message group read all or almost all the messages [47]. The messages from the Text4PTSI were based on CBT and trauma therapy principles and crafted by mental health professionals along with service users, thus encouraging the participants to read and reflect on the texts. The results also may also explain why 76% at least sometimes read the messages more than once. When an intervention is acceptable and effective, patients are more likely to accept the treatment and desire to continue with the intervention [48]. Participants in our study reported being satisfied with the frequency of text messages (93%). While according to the literature, user ratings of a given service may provide limited evidence about the value of an intervention (eg, social desirability) [49], when individuals are content with the frequency and time they receive text messages, it increases their chance of engaging with the text, with approximately 91% of texts read within the first few minutes of receipt [50].

### Limitations

There are several limitations to the study, which need to be considered when interpreting the results. First, the web-based questionnaire to measure the satisfaction and perception of the study participant was not a validated instrument and may limit the confidence of user satisfaction rates. Second, our sample was not randomized; it would have been ideal to include a control group to compare Text4PTSI subscribers’ and nonsubscribers’ satisfaction, perception, and impact of the program on their mental health. Third, the number of first responders who subscribed to the Text4PTSI program was less than 10% of the projected 5000 subscribers indicated in the published protocol [33]. Challenges associated with the adoption and promotion of the program by first responder organizations in Alberta, despite the initiative being a government-funded program, contributed to the low uptake overall.

Fourth, the sample size of participants who completed all follow-up assessments was fairly small, which may be attributed to participants who still needed to complete the survey and those providing incomplete responses. Out of the 131 subscribers, only 81 responded to the survey. A total of 393 responses were expected during the follow-up; however, 100 survey responses were provided by participants. In addition, the low sample size may be a result of the web-based nature of the surveys. Surveys delivered via SMS text messages are less likely to retain participants for follow-up assessment than paper-based surveys [51].

Notwithstanding these limitations, the findings from our study suggest that text-based health interventions designed for PSP have high satisfaction rates and may be beneficial to improve quality of life and help bridge the gap in seeking mental health services.

### Conclusions

Our results highlighted findings relating to the rationale for PSP’s desire to subscribe to supportive text messaging programs. The study generated important insights into the self-reported perception of supportive text messages that improved quality of life and overall mental well-being; coping with anxiety, depression, and loneliness; and being connected to a support network. These results are derived from a few samples who completed the follow-up survey; more research is needed to better understand what is happening with nonrespondents. Additionally, future randomized controlled studies are recommended to comprehensively assess the impact, user satisfaction, perception, and receptivity of the SMS text messaging intervention on PSP and can be scaled up to other occupations, and to better understand the reasons why study participants may opt not to respond to the study surveys that could assess their well-being and satisfaction with the service. Furthermore, research needs to explore possible ways that could be used to reach those who do not respond to the surveys. SMS text messages have fewer character limits, which may improve the participants’ desire to read.

The results of this study provide additional evidence for scale up and spread of supportive text message interventions for PSP and other stressful occupations.

## Abbreviations

CBT: cognitive behavioral therapy
iCBT: internet cognitive behavioral therapy
PSP: public safety personnel

## Data Availability

Data associated with this study will be made freely available upon reasonable request to the corresponding author.
